# Preferences for Social Media Vaccination Messaging

**DOI:** 10.1001/jamanetworkopen.2026.2284

**Published:** 2026-03-18

**Authors:** Lucía Abascal Miguel, Alison B. Comfort, Alicia R. Riley, Gilberto Lopez, Janelli Vallin, Anna E. Epperson, Nadia Diamond-Smith

**Affiliations:** 1 Institute for Global Health Sciences, University of California, San Francisco, San Francisco; 2 Department of Obstetrics, Gynecology and Reproductive Sciences, University of California, San Francisco, San Francisco; 3 Department of Sociology, University of California, Santa Cruz, Santa Cruz; 4 University of California Santa Cruz Institute for Social Transformation, University of California, Santa Cruz, Santa Cruz; 5 School of Transborder Studies, Arizona State University, Tempe; 6 Department of Psychological Sciences, University of California, Merced, Merced; 7 Department of Epidemiology and Biostatistics, University of California, San Francisco, San Francisco

## Abstract

**Importance:**

Social media is a dominant source of health information, including vaccine information, but little is known about which message characteristics audiences prefer when deciding what social media content to engage with and about how to measure these preferences.

**Objective:**

To quantify the attributes of existing social media vaccination posts that are associated with the preference for and confidence in vaccination-related content.

**Design, Setting, and Participants:**

This cross-sectional study used a combination of the discrete choice experiment (DCE) and the Swiss tournament approaches to evaluate preferences, which were collected in an online survey between March and May 2024. Participants (aged ≥18 years) from a previous California-based COVID-19 study were recontacted through social media between January and August 2024. Participants completed the DCE and Swiss tournament test.

**Exposures:**

Participants viewed pairs of vaccination-related social media posts that varied systematically by attributes, such as messenger, source, tone, artwork type, age group of messenger, and topic.

**Main Outcomes and Measures:**

Participants selected which post they were most likely to engage with (like, share, or comment). Preferences were analyzed using a conditional logit model to estimate relative importance of post attributes.

**Results:**

Among 243 participants (mean [SD] age, 36.4 [12.4] years; 127 males [52.5%]), 153 (63.0%) reported favorable attitudes toward vaccines in general, 167 (68.7%) toward influenza vaccines, and 117 (48.2%) toward COVID-19 vaccines. Daily social media use was common (228 [93.8%]), and most participants preferred visual content, such as pictures (206 [84.8%]) or short videos (184 [75.7%]). In the DCE, social media vaccination posts from public health agencies such as California Department of Public Health (adjusted odds ratio [AOR], 1.80; 95% CI, 1.57-2.07) and University of California San Francisco (AOR, 1.67; 95% CI, 1.21-2.29) and posts depicting health care workers (AOR, 1.28; 95% CI, 1.12-1.47) or older adults (AOR, 1.48; 95% CI, 1.22-1.80) were more likely to be preferred. Humorous tone was associated with reduced preference (AOR, 0.45; 95% CI, 0.36-0.57), whereas COVID-19 (AOR, 2.35; 95% CI, 1.95-2.87) and influenza (AOR, 1.78; 95% CI, 1.54-2.06) topics were associated with increased likelihood for preference. Artwork type showed no significant association with preference.

**Conclusions and Relevance:**

This cross-sectional study found that participants preferred social media–based vaccination posts that were factual, featured health care professionals, and were sourced from public health organizations. Understanding communication preferences through innovative experimental methods can inform the design of digital public health communications.

## Key Points

**Question:**

Which characteristics of social media vaccination posts do audiences prefer when deciding which content to engage with?

**Findings:**

In this cross-sectional study of 243 adults in California, social media vaccination posts that were factual and from trusted public health organizations and health care workers were significantly more likely to be preferred than humorous or unsourced posts. The artwork type of a post showed no significant association with preference.

**Meaning:**

Findings from this study suggest that innovative experimental methods, such as discrete choice experiment, can inform the design of digital public health communication.

## Teaser

This cross-sectional study identifies the attributes of vaccination-related posts on social media associated with audience engagement.

## Introduction

Rising vaccine hesitancy presents substantial challenges to public health.^1^ Influenza and COVID-19 are leading causes of hospitalizations and deaths.^2,3^ Yearly vaccination is one of the most effective ways to prevent severe adverse outcomes.^4,5,6^ Despite these recommendations, vaccination coverage remains low in California. Only 13% of eligible individuals received the updated COVID-19 vaccine in 2024, and influenza vaccine uptake has declined since the pandemic to less than 50% statewide.^7,8^

Social media has emerged as a powerful public health tool, enabling rapid and interactive communication that reaches diverse audiences.^9,10^ Platforms such as Facebook and Instagram (Meta Platforms Inc) allow health organizations to engage the public and change vaccination attitudes. Social media interventions have shown considerable success in boosting vaccination rates by increasing vaccine knowledge and confidence.^11,12,13^ Despite widespread use of social media for vaccine education, few studies have identified the specific characteristics of social media–based communications that people prefer.

Vaccine messages from trusted messengers can benefit vaccine acceptance, increasing favorable beliefs and reducing adverse attitudes.^14,15^ Visual content, including videos and infographics, has also been shown to be more effective than text-based posts in promoting vaccine literacy, and videos featuring health care practitioners can increase vaccination intentions among unvaccinated adults.^16,17^ Designing effective messages remains complex, as factors such as tone, level of detail, visual elements (eg, cartoons vs real images), and portrayal of trusted messengers all affect engagement. In the context of widespread misinformation, ensuring that messages are accurate and trustworthy is critical. Despite growing evidence on social media’s role in shaping vaccine attitudes, few US studies have systematically identified which social media message characteristics most likely affect preferences for COVID-19 or influenza vaccination content.

Discrete choice experiments (DCEs) provide a structured way to assess preferences by comparing different attributes and eliciting ordered preferences. This method is widely used to quantify patient and stakeholder preferences and can inform intervention design by identifying which attributes are most valued.^18,19,20^ However, it has been used far less often to study public health messaging, with which DCE could help by identifying user preferences and improving the design of social media–based campaigns. In this study, we aimed to quantify the attributes of existing social media vaccination posts that affect the preference for and confidence in vaccination-related content.

## Methods

We used a combined DCE and Swiss tournament design to evaluate survey-collected preferences for vaccination-related social media posts. The DCE estimated preferences for specific post attributes, generating insights that could inform the design of public health communication on social media. The Swiss tournament structure assessed overall preferences for complete posts. Participants underwent multiple rounds of paired comparisons in which posts were adaptively matched based on prior performance, allowing higher-ranked posts to advance without requiring exhaustive comparisons. While DCE isolated attribute-level preferences, the Swiss tournament approach efficiently identified the most preferred posts overall. The University of California, San Francisco (UCSF) Institutional Review Boards approved this cross-sectional study. All participants provided informed consent electronically before participation. We followed the Strengthening the Reporting of Observational Studies in Epidemiology (STROBE) reporting guideline.

### Preliminary Work and Post Selection

We identified relevant attributes and levels for the DCE through a literature review, consultations with the Community Advisory Board we formed for this project, health messaging experts, and findings from an initial survey. Attributes represent key features of the social media posts that might affect preference, whereas levels reflect variations within each attribute.

We then selected 16 existing vaccination-related social media posts developed by public health organizations that reflected these attributes. Posts were categorized by the following attributes: artwork type (levels: cartoon or real photographs), messenger (levels: community members or health care workers), source (levels: unsourced, Centers for Disease Control and Prevention [CDC], California Department of Public Health [CDPH], or UCSF), tone (levels: factual, funny, or informative), age group of messenger (levels: all ages, midlife adults, or older adults), and topic (levels: general vaccination, COVID-19, or influenza) (Box). Two posts were generated using DALL-E (OpenAI). CDC posts were obtained from the agency’s public resource library, while CDPH and COVIDLatino posts were sourced directly from those organizations. We developed the UCSF and unsourced posts in collaboration with a graphic designer.

Box. **Health Post Attributes**

**Artwork Type**

Cartoon
Real photographs

**Messenger**

Community members
Health care workers

**Source**

Unsourced
Centers for Disease Control and Prevention
California Department of Public Health
University of California San Francisco

**Tone**

Factual
Funny
Informative

**Age Group of Messenger**

All ages
Midlife adults
Older adults

**Topic**

General vaccination
COVID-19
Influenza

Due to copyright restrictions, we displayed only images for which we had explicit permission to reproduce, including materials created by our research team and by COVIDLatino. Posts incorporating stock images were modified to ensure faces were not identifiable. Posts created using artificial intelligence were not included, as no clear guidelines regarding their reproduction and copyright have been established.

The complete set of posts used in the study, along with their full attribute categorization and the DCE design matrix, is provided in the eTable in Supplement 1. Because we used existing social media posts rather than experimentally constructed, orthogonal vignettes, attributes were not fully independent by design and may have co-occurred across posts.

### Sampling and Recruitment

Participants in the online survey were selected from a larger pool of individuals who had previously participated in a related COVID-19 study and agreed to be recontacted for future research. The parent study used Facebook and Instagram to recruit adults (aged ≥18 years) living in California from January to August 2024. From that initial sample, we conducted race and ethnicity–stratified random sampling to reflect California’s population distribution based on the most recent US Census (2020). Participants were contacted via email, receiving a 1-time-use link to access the survey. Of the individuals who opened the invitation email and completed the survey, only those with finished surveys were included in our sample. Participants received a $30 electronic gift card upon completion. A participant flow diagram is provided in eFigure 2 in Supplement 1.

### Sample Size Calculation

The sample size for the DCE was calculated using the Johnson and Orme rule of thumb. It recommends a minimum sample size based on this formula: N>500 × c/t × a, where c is the largest number of levels for any attribute (4 in this study), t is the number of choice tasks per respondent (8 choice sets), and a is the number of alternatives per choice task (2). Using these parameters, the minimum recommended sample size was 125 respondents.^21^

### Survey Design

We conducted the survey using Qualtrics.^22^ After consenting, participants were asked to provide basic demographic information, including age, gender, race and ethnicity, and educational level. Self-reported race and ethnicity (American Indian or Alaska Native, Asian, Black or African American, Hispanic or Latino, White, other [Middle Eastern or North African, Native Hawaiian or Other Pacific Islander, and unspecified]) were collected to reflect California's race and ethnicity distribution. Participants were asked about their general attitudes, beliefs, and behaviors toward vaccines (specifically COVID-19 and influenza vaccines) and their social media use, including how often they encountered and shared health-related content on these platforms.

Participants reviewed pairs of vaccination-related social media posts and selected the ones they were more likely to like, comment on, or share. The survey included 16 posts arranged in a Swiss tournament format, a nonelimination structure with 6 rounds of pairwise comparisons for a total of 48 comparisons (full structure is shown in eFigure 1 in Supplement 1). In the first round, posts were randomly paired; posts selected as winners from each pair advanced to face other winners in subsequent rounds, while the nonselected posts were paired together. This process continued until the sixth (final) round, where the top posts were compared to identify overall preference. The Swiss tournament structure served 2 purposes: first-round comparisons provided randomly paired choices for the DCE, while later rounds ranked complete posts based on overall preference rather than estimated attribute-level associations.

### Statistical Analysis

To analyze the Swiss tournament results, we calculated the proportion of times each post was selected as the preferred options across individual pairings. The DCE analysis was restricted to the first round of 8 randomly paired choice tasks completed by each participant. Although participants continued to make selections in subsequent Swiss tournament rounds, these later choices involved adaptive pairing and were not included in the DCE; instead, they were analyzed descriptively to generate overall post rankings. This approach allowed the DCE to estimate attribute-level preferences, while the Swiss tournament rankings summarized overall preference for complete posts. In the DCE, attributes were treated as categorical dummy variables, each with a unique identification number.

We applied a conditional logit model (McFadden choice model) to account for repeated choices made by the same participant.^23^ Because each participant completed multiple choice tasks, the model conditions on the respondent, comparing alternatives within each choice set rather than across individuals. All post attributes (Box) were included simultaneously in the statistical model to estimate the association of each attribute with post selection. A 2-sided *P* < .05 was set to indicate statistically significant preferences. Using the Stata cmset command (StataCorp LLC), we structured the dataset for choice modeling by defining the choice tasks, participant identification numbers, and available alternatives, linking individual choices to specific attributes.^24^ The conditional logit model then estimated the probability of selecting a given option based on its attributes, with the sign of the choice coefficient indicating whether the attribute had a favorable or adverse role in preference. Model estimates were interpreted as associations reflecting relative preferences for post attributes rather than causation of individual message features.

For the model, the dependent variable was the participant’s choice (a binary measure for whether the post was selected), while the independent variables were the post attributes (Box). Each post attribute was treated as an independent variable in the analysis. We also included participant attributes such as age, gender, and race and ethnicity.

## Results

A total of 243 adults (mean [SD] age, 36.4 [12.4] years) residing in California were included in the study. This sample was diverse in gender (108 females [44.2%], 127 males [52.5%], 2 transgender females [0.8%], 1 transgender male [0.4%], 1 nonbinary or gender fluid [0.4%], 4 unspecified or unanswered [1.7%]), race and ethnicity (40 American Indian or Alaska Native [16.5%], 24 Asian [9.9%], 24 Black or African American [9.9%], 106 Hispanic or Latino [43.6%], 85 White [34.9%], 4 other [1.7%]), and educational level. Most participants had a Bachelor’s degree (122 [50.4%]) and were born in the US (219 [90.5%]) (Table 1).

**Table 1.** Participant Characteristics

Characteristic	Participants, No. (%) [n = 243]
Age, mean (SD), y	36.4 (10)
Gender	
Male	127 (52.5)
Female	108 (44.2)
Transgender male	1 (0.4)
Transgender female	2 (0.8)
Nonbinary or gender fluid	1 (0.4)
Unspecified or prefer not to answer	4 (1.7)
Race and ethnicity^a^	
American Indian or Alaska Native	40 (16.5)
Asian	24 (9.9)
Black or African American	24 (9.9)
Hispanic or Latino	106 (43.6)
White	85 (34.9)
Other^b^	4 (1.7)
Educational level	
<High school	3 (1.2)
High school diploma	27 (11.2)
Some college or associate degree	33 (13.6)
Bachelor’s degree	122 (50.4)
Master’s or professional degree	38 (15.7)
Prefer not to answer	20 (8.2)
Born in the US	219 (90.5)

^a^ Race and ethnicity were self-reported, and the categories were not mutually exclusive; participants could select multiple identities.

^b^ Other category included Middle Eastern or North African, Native Hawaiian or Other Pacific Islander, and unspecified other.

### Vaccine Attitudes and Social Media

Participants in our sample generally held favorable attitudes toward vaccines, with the highest support observed for general vaccines (153 [63.0%] rating very positive) and influenza vaccines (167 [68.7%] rating very positive). COVID-19 vaccines received slightly lower positive ratings, with 117 (48.2%) choosing very positive and 22 (9.0%) expressing somewhat negative and very negative views. Social media use was high, with 228 participants (93.8%) using it every day and 130 participants (53.5%) sharing content at least once a day. Health-related content was widely encountered. Participants reported receiving health-related messages multiple times a day (84 [34.6%]), at least once a day (47 [19.3%]), or a few times a month (88 [36.2%]). Only 23 participants (9.5%) said they rarely or never saw health-related messages.

Data from the survey questions showed that, in terms of preferred social media post formats, participants had a clear preference for visual content: 206 participants (84.8%) selected static pictures, and 184 (75.7%) favored short videos. In contrast, text-based content had moderate appeal, with 139 (57.2%) selecting text posts, while only 63 (25.9%) preferring longer videos. Close family and friends emerged as the most trusted messengers for health-related social media content; specifically, 190 participants (78.2%) trusted friends, 187 (76.9%) trusted parents, 179 (73.7%) trusted siblings, and 178 (73.3%) trusted partners as sources for sharing health information. Health care practitioners were also trusted messengers, chosen by 165 participants (67.9%), and community leaders were trusted by 95 participants (39.1%).

### Swiss Tournament

The Swiss tournament results described overall preferences for complete social media posts across multiple rounds of pairwise comparisons. The most frequently selected post was a CDPH post featuring a young woman displaying an adhesive bandage on her arm, promoting National Influenza Vaccination Week (post 7) (eTable in Supplement 1), which won 23% of tournaments. In second place, winning 15% of tournaments, was a CDC post highlighting serious influenza-related health risks, including heart complications and intensive care unit admissions (post 15). The third place, another CDPH post and selected in 14% of tournaments, depicted an older man flexing his arm to show an adhesive bandage with a message encouraging COVID-19 vaccination (post 4).

### Discrete Choice Experiment

The DCE results quantified preferences for individual post attributes using first-round, randomly paired comparisons. We found that all post attributes, except for artwork type, had a significant association with preference (Table 2). For the source attribute, posts from the CDPH were more likely to be preferred over unsourced posts (adjusted odds ratio [AOR], 1.80; 95% CI, 1.57-2.07). Posts from UCSF also showed a higher likelihood of being selected (AOR, 1.67; 95% CI, 1.21-2.29), while CDC posts had lower preference (AOR, 0.81; 95% CI, 0.64-1.01). For the messenger and age group attributes, posts featuring health care workers were more likely to be chosen over those featuring community members (AOR, 1.28; 95% CI, 1.12-1.47). Posts depicting older adults were more likely to be preferred (AOR, 1.48; 95% CI, 1.22-1.80) as were posts featuring midlife adults (AOR, 1.38; 95% CI, 1.20-1.59), compared with posts depicting other age groups. In terms of the tone attribute, humorous or funny posts were less likely to be chosen than factual posts (AOR, 0.45; 95% CI, 0.36-0.57). Informative posts were not significantly more likely to be preferred compared with posts that stated scientific facts (AOR, 1.09; 95% CI, 0.92-1.29). Finally, for the topic attribute, COVID-19–related posts had a substantially higher likelihood of being chosen (AOR, 2.35; 95% CI, 1.95-2.87), as did posts about influenza (AOR, 1.78; 95% CI, 1.54-2.06), than posts on general vaccination.

**Table 2.** Association Between Social Media Post Preferences and Attributes of Health Posts

Attributes	AOR (95% CI)^a^	*P* value
Artwork type		
Cartoon	1 [Reference]	NA
Real photographs	1.06 (0.91-1.24)	.41
Source		
Unsourced	1 [Reference]	NA
CDC	0.81 (0.64-1.01)	.06
CDPH	1.80 (1.57-2.07)	<.001
UCSF	1.67 (1.21-2.29)	.002
Messenger		
Community members	1 [Reference]	NA
Health care workers	1.28 (1.12-1.47)	<.001
Age group of messenger		
All ages	1 [Reference]	NA
Older adults	1.48 (1.22-1.80)	<.001
Midlife adults	1.38 (1.20-1.59)	<.001
Tone		
Factual	1 [Reference]	NA
Funny	0.45 (0.36-0.57)	<.001
Informative	1.09 (0.92-1.29)	<.001
Topic		
General vaccination	1 [Reference]	NA
COVID-19	2.35 (1.95-2.87)	<.001
Influenza	1.78 (1.54-2.06)	<.001

Abbreviations: AOR, adjusted odds ratio; CDC, Centers for Disease Control and Prevention; CDPH, California Department of Public Health; NA, not applicable; UCSF, University of California San Francisco.

^a^ Models represent conditional logit models adjusting for participant age, gender, race and ethnicity, and educational level.

## Discussion

Using a combination of a Swiss tournament and DCE design, this cross-sectional study examined preferences among Californians for messaging characteristics in social media posts about vaccination. Participants preferred factual messages, over humorous or unsourced messages, and posts that were specific to COVID-19 or influenza. Posts featuring health care workers and older adults, rather than community members or mixed-age groups, were preferred. Posts with an identifiable source were preferred over unsourced posts, and among sourced posts, those attributed to local health institutions (CDPH and UCSF) were preferred over those attributed to the CDC.

The finding that health care workers are preferred as messengers is consistent with prior work showing that health care professionals are often perceived as credible messengers in health communication, while also highlighting substantial variation across institutional sources.^25,26^ While humorous content has been found in some studies to be preferential in certain subgroups, such as among American Indians or Alaska Native individuals, this preference did not generalize to our sample.^27,28^ Our findings suggest that while humor may resonate in specific contexts, survey participants favored straightforward, factual information. We found no clear preference for cartoon or animated images vs real photographs, which contrasts with studies that found that depictions of real people were more effective in building trust in First Nations communities in Australia.^29^ This contrasting finding suggests that different populations may respond differently to visual styles, which has implications for campaign costs, as photographs can be more expensive to produce than cartoons. Further research is needed to explore these preferences across different demographic groups.

Given the volume of vaccine information, including misinformation, on social media, it is critical that public health messaging is evidence based and resonates with intended audiences. In this study, participants preferred COVID-19 and influenza posts that were factual, clearly sourced from public health organizations, and featured health care workers or older adults, suggesting that credibility and perceived authority are central to message preference. At the same time, preferences varied by message characteristics, highlighting the importance of empirically testing content rather than relying on assumptions about what audiences prefer. Our findings highlight the value of using structured, preference-based methods, such as DCEs, to systematically evaluate message features and inform the design of more effective, evidence-driven public health communication strategies.

The findings from this study will inform the design of a social media-based study on COVID-19 and influenza vaccination messaging in California, evaluating the association of such messaging with vaccine intentions and uptake. We will also share our insights with key stakeholders to enhance public health messaging strategies. Assessing audience preferences prior to campaign design may improve engagement and strengthen vaccination-related knowledge.

### Limitations

This study has several limitations. First, unlike typical DCEs that use experimentally balanced attributes, we used existing posts developed for actual campaigns. As a result, attributes were not fully orthogonal and may have co-occurred across posts, introducing the potential for residual confounding and reflecting a trade-off between experimental control and ecological validity.

Second, because this study relied on stated preferences collected through a survey, responses may have been affected by social desirability bias. Participants may have been more likely to select posts perceived as responsible or credible rather than content that might attract attention through humor.

Third, the forced-choice design does not replicate social media environments, and findings reflect preference rather than observed engagement behavior. However, such designs are well suited to eliciting comparative preferences and trade-offs across message attributes and are intended to complement and inform A/B testing of live campaigns. In addition, the outcome focused on intended favorable engagement (liking or sharing) and did not capture other forms of adverse interaction.

Fourth, the sample was drawn from a prior study and may not fully represent the broader California population, particularly with respect to educational level. This sample was not large enough to allow for subgroup analysis across specific groups, limiting our ability to draw conclusions about demographic-specific preferences or to examine interactions between attributes and participant characteristics. Finally, as DCEs are inherently associative rather than causal, our findings should be interpreted as preferences and not causation of behavior. Future studies should consider using randomized, balanced attributes and larger, more diverse samples to strengthen the robustness of findings.

## Conclusions

In this cross-sectional study of adults in California, participants preferred vaccination-related social media posts that were factual, sourced from public health organizations, and featured health care professionals or older adults. Posts focused on COVID-19 and influenza were more likely to be selected than general vaccination messages, while humorous tone was less preferred. These findings illustrate how experimental preference-based methods can inform the design of digital public health messaging.

## Notes

Supplement 1. **eTable.** Discrete Choice Experiment Post Profiles and Attribute Coding **eFigure 1.** Swiss Tournament Structure **eFigure 2.** Participant Flow Diagram

Supplement 2.
Data Sharing Statement
