# Supplementary material for: Preferences for Social Media Vaccination Messaging
Source: JAMA Netw Open. 2026 Mar 18;9(3):e262284. doi: 10.1001/jamanetworkopen.2026.2284 (PMC13000627; doi:10.1001/jamanetworkopen.2026.2284)
Supplement: Supplement 1. — eTable. Discrete Choice Experiment Post Profiles and Attribute Coding eFigure 1. Swiss Tournament Structure eFigure 2. Participant Flow Diagram [file jamanetwopen-e262284-s001.pdf]

Supplemental Online Content

Abascal Miguel L, Comfort AB, Riley AR, et al. Preferences for social media vaccination messaging. *JAMA Netw Open*. 2026;9(3):e262284. doi:10.1001/jamanetworkopen.2026.2284

eTable. Discrete Choice Experiment Post Profiles and Attribute Coding

eFigure 1. Swiss Tournament Structure

eFigure 2. Participant Flow Diagram

This supplemental material has been provided by the authors to give readers additional information about their work.

© 2026 Abascal Miguel L et al. *JAMA Network Open*

Post	Artwork Type	Messenger Depicted	Source Attribution	Tone	Format	Age Group Depicted	Topic
Post 1. AI developed Cartoon illustration of a diverse group of community members of different ages and ethnicities standing together with bandages on their arms under a banner reading "Let's Vaccinate!" Developed using OpenAI's Dalle	Cartoon illustration	Community members	None (unsourced)	Factual	Static image	All ages	General vaccination
Post 2. AI developed Cartoon-style illustration of a diverse group of community members of different ages and ethnicities standing together and smiling, holding signs that read "Let's Get Vaccinated" and "Protect Yourself." Developed using OpenAI's Dalle	Cartoon illustration	Community members	None (unsourced)	Factual	Static image	All ages	General vaccination
Post 3. Pixel-art, video-game–style graphic showing a character powering up to defeat COVID-19 using a vaccine syringe and a face mask, with the headline "Power Up! Defeat COVID-19 with a vaccine and a good mask." Original post available at: https://share.google/images/rYfYWikg0vx0hJUrr	Cartoon illustration	Community members	CDPH	Humorous	Static image	Not specified	COVID-19
Post 4. CDPH Graphic featuring a picture of an older adult crossing their arms in an "X" gesture over their chest, with text reading "Get the Updated COVID-19 Vaccine."	Real photographs	Community members	CDPH	Informative	Static image	Older adults	COVID-19
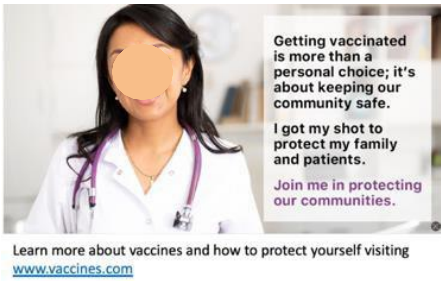	Real photographs	Health worker	None (unsourced)	Factual	Static image	Midlife adults	General vaccination

© 2026 Abascal Miguel L et al. *JAMA Network Open*

Post 6. Graphic illustration announcing "This week is National Influenza Vaccination Week!" showing a young adult with a bandage on their upper arm and logos for Ad Council, AMA, and CDC. Original post available at: https://www.cdc.gov/flu-resources/php/resources/protect-family-fb.html	Cartoon illustration	Community members	CDC	Informative	Static image	Midlife adults	Influenza
Post 7. Real photograph of a woman showing a bandage on her upper arm next to text reading "National Influenza Vaccination Week — December 6–12," with a call to action to visit MyTurn.ca.gov.	Real photographs	Community members	CDPH	Informative	Static image	Midlife adults	Influenza
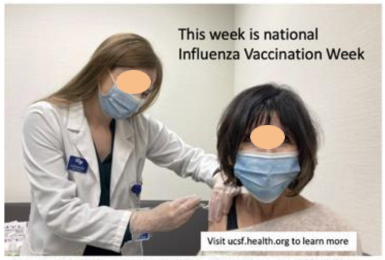<br><br>Learn more about vaccines and how to protect yourself visiting www.vaccines.com	Real photographs	Health worker	UCSF	Informative	Static image	Older adults	Influenza
Post 9. Graphic illustration titled "Flu Q&A," featuring a stylized raised fist labeled "FLU" and bullet points explaining how the flu spreads. Original post available at: https://www.cdc.gov/flu-resources/php/resources/index.html#cc-widget-36cb (under social media graphics)	Cartoon illustration	None clearly depicted	CDC	Factual	Static image	All ages / unspecified	Influenza
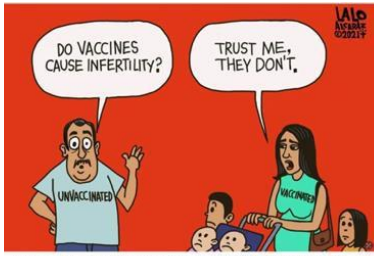<br><br>Learn more about vaccines and how to protect yourself visiting www.vaccines.com	Cartoon illustration	Community members	None (unsourced)	Humorous	Static image	All ages / unspecified	General vaccination

© 2026 Abascal Miguel L et al. *JAMA Network Open*

Post 11. CDPH cartoon-style illustration of two scientists working in a laboratory with overlaid text reading “Long COVID can put your job and your life on hold” and “Prevent long COVID. Get vaccinated and boosted.” Original post available at: https://www.cdph.ca.gov/Programs/CCDPHP/DEODC/OHB/Pages/LongCOVIDTools.aspx	Cartoon illustration	Health worker	CDPH	Factual	Static image	All ages / unspecified	COVID-19
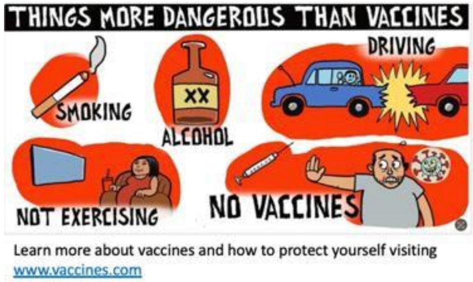	Cartoon illustration	Community members	None (unsourced)	Humorous	Static image	Not specified	General vaccination
Post 13. Real photograph of a female doctor sitting down, with overlaid text reading “Flu vaccines are available in your community and often free as they are covered by insurance.” Logos for Ad Council and CDC. Original post available at: https://getmyflushot.org/	Real photographs	Health worker	CDC	Informative	Static image	Midlife adults	Influenza
Post 14. Real photograph of a person wearing a winter hat and scarf and holding a tissue to their face, with overlaid text reading “Don’t let flu, RSV, or COVID-19 happen to you” and “Slow the spread.” With CDPH logo. Original post available at: https://www.calhr.ca.gov/2024/11/13/cold-and-flu-season-advisory-message-from-calhhs-secretary-kim-johnson/	Real photographs	Community members	CDPH	Humorous	Static image	Midlife adults	General vaccination
Post 15. Graphic illustration summarizing findings from a study of more than 80,000 adults hospitalized with flu, stating that one in eight patients had sudden heart complications and that almost one-third required ICU care and 7% died. The post includes text noting that flu vaccination reduces the risk of heart complications and death. CDC logo. Original post	Cartoon illustration	None clearly depicted	CDC	Factual	Static image	Not specified	Influenza

© 2026 Abascal Miguel L et al. *JAMA Network Open*

available at: https://www.cdc.gov/flu-resources/php/resources/index.html#cc-widget-36cb (under social media graphics)							
Post 16. Real photograph of a health worker administering a vaccine to an older adult patient in a clinical setting, with overlaid text reading "If you're 65+, it's important to get a flu shot this season." CDPH logo. Original post available at: https://www.cdph.ca.gov/Programs/OPA/Pages/Communications-Toolkits/my-turn-flu.aspx	Real photographs	Community members	CDPH	Informative	Static image	Older adults	Influenza

eTable. Discrete Choice Experiment Post Profiles and Attribute Coding

Notes:

1. Posts represent real social media materials previously used in public health communication campaigns. Attributes were coded based on visible visual and textual elements. Posts were not experimentally constructed to achieve orthogonality; therefore, attributes may co-occur across profiles. "Unsourced" indicates the absence of explicit institutional attribution within the post. This table is provided to document the stimuli used and the attribute structure underlying the discrete choice experiment.
2. Due to copyright restrictions, we are only able to display images for which we had explicit permission to reproduce. These include materials created in-house by the research team and by COVIDLatino. For posts that incorporated stock images, the visuals were modified so that individuals are not identifiable. Posts created using artificial intelligence were not included, as clear guidelines regarding their reproduction and copyright status are not yet established.
3. www.vaccines.com is not a real website and was just used in the image as an example of a call to action, participants couldn't click or interact with it.

eFigure 1. Swiss Tournament Structure

Posts selected as preferred (“wins,” shown with green arrows) and not preferred (“losses,” shown with red arrows) advanced to subsequent tournament rounds. In later rounds, posts were adaptively re-paired based on prior performance, such that posts with similar win–loss records were compared against each other.

Boxes labeled “**W**” and “**L**” indicate posts that won or lost in the preceding round, respectively. Posts that accumulated enough losses exited the tournament (“Out”), whereas higher-performing posts continued to advance (“In”). Posts that remained “In” entered a final bracketed phase to identify the top-ranked finalists.

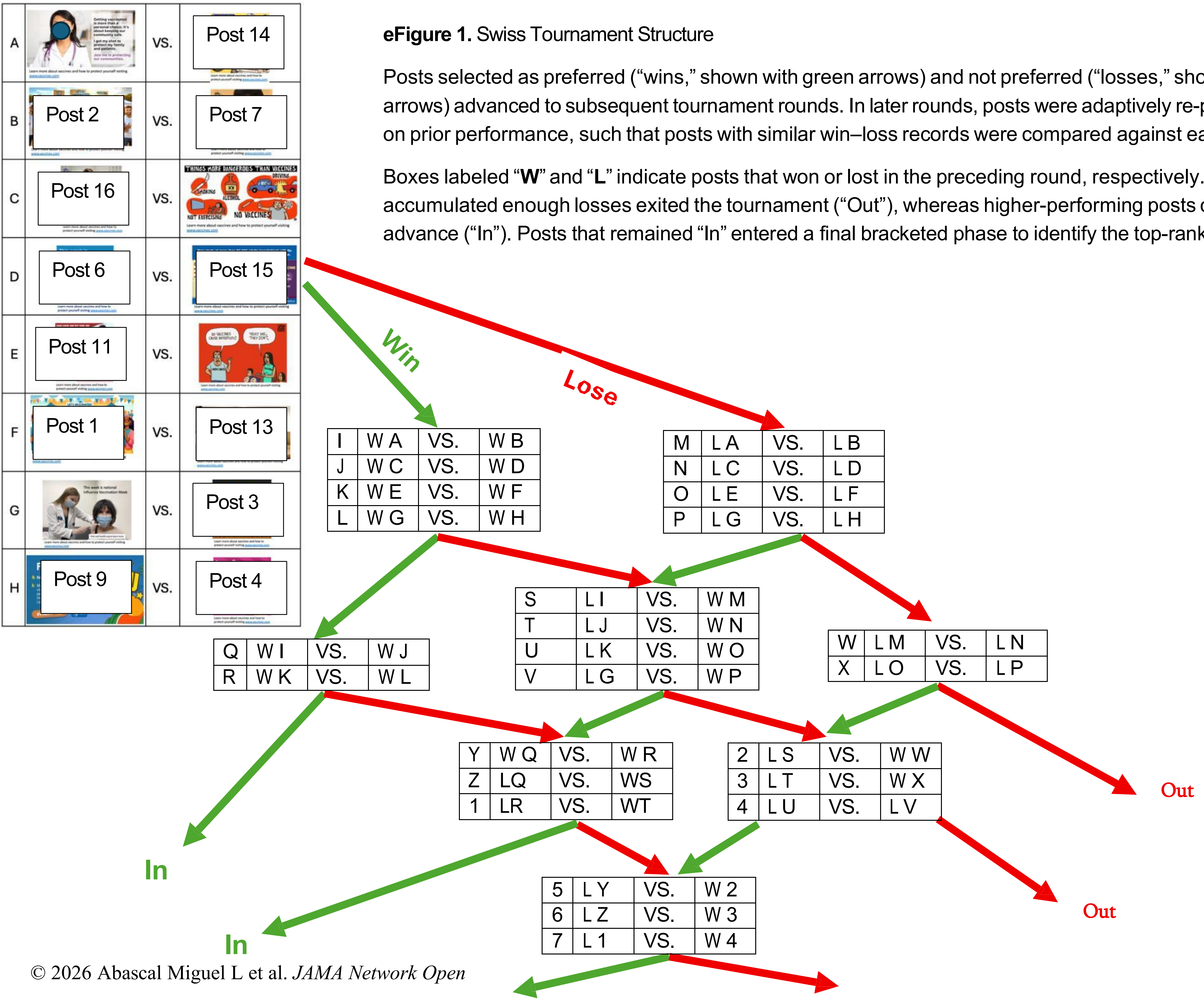

© 2026 Abascal Miguel L et al. *JAMA Network Open*

© 2026 Abascal Miguel L et al. *JAMA Network Open*

eFigure 2. Participant Flow Diagram

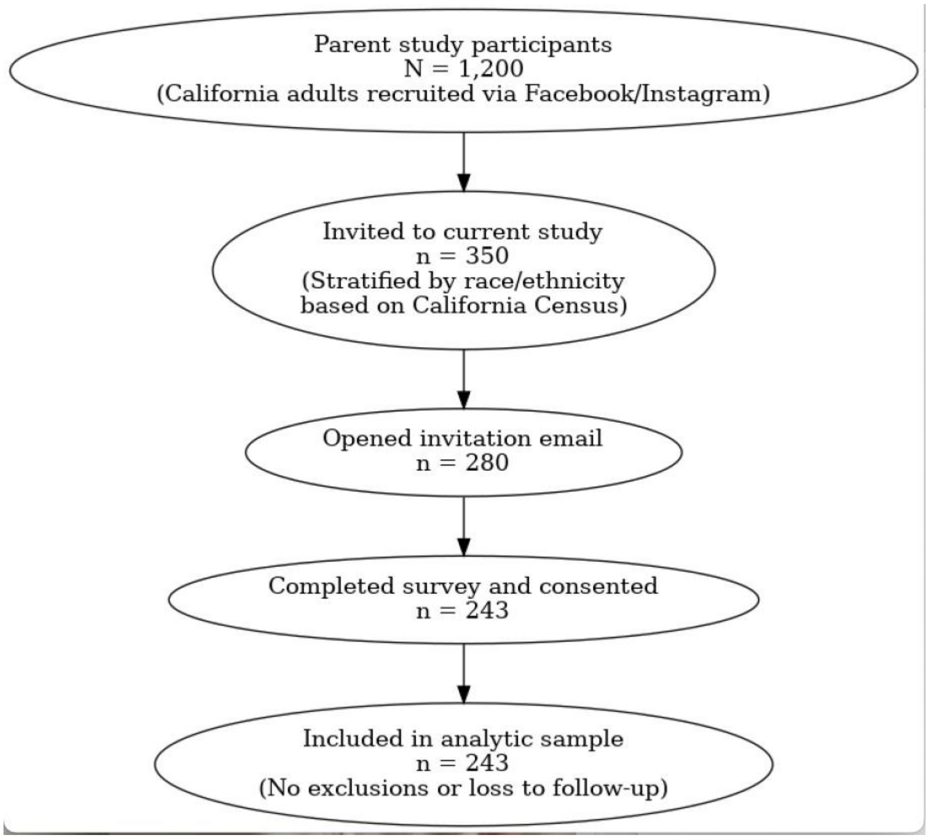

© 2026 Abascal Miguel L et al. *JAMA Network Open*
