# Supplementary material for: Preferences for Social Media Vaccination Messaging
Source: JAMA Netw Open. 2026 Mar 18;9(3):e262284. doi: 10.1001/jamanetworkopen.2026.2284 (PMC13000627; doi:10.1001/jamanetworkopen.2026.2284)
Supplement: Supplement 2. — Data Sharing Statement [file jamanetwopen-e262284-s002.pdf]

Data Sharing Statement

Miguel. Preferences for Social Media Vaccination Messaging. *JAMA Netw Open*. Published March 18, 2026. doi:10.1001/jamanetworkopen.2026.2284

Data
Data available: Yes
Data types: Deidentified participant data
How to access data: lucia.abascal@ucsf.edu
When available: With publication

Supporting Documents
Document types: Informed consent form
How to access documents: lucia.abascal@ucsf.edu
When available: With publication

Additional Information
Who can access the data: researchers whose proposed use of the data has been approved
Types of analyses: For any purpose
Mechanisms of data availability: With a signed data access agreement
